# Group size experiences with enhanced pre- and postnatal development studies in the long-tailed macaque (*Macaca fascicularis*)

**DOI:** 10.5194/pb-7-1-2020

**Published:** 2020-03-11

**Authors:** C. Marc Luetjens, Antje Fuchs, Ann Baker, Gerhard F. Weinbauer

**Affiliations:** 1 Covance Preclinical Services GmbH, Kesselfeld 29, 48163 Münster, Germany; 2 Covance Laboratories, Madison, WI, USA

## Abstract

Enhanced pre- and postnatal development (ePPND) studies have become the default developmental toxicity test for biopharmaceuticals if nonhuman primates represent the relevant species. Spontaneous pregnancy losses and infant deaths can be significant in macaques such as long-tailed macaques. The International Council for Harmonisation of Technical Requirements for Pharmaceuticals for Human Use (ICH) guideline S6(R1) states that pregnancy outcome can be judged also by the normogram-based variability of reference data according to a publication by Jarvis et al. (2010) defining a study as valid with six to eight live infants in the control group on postnatal day 7 (PND7). Since the release of ICH S6(R1) (2011), ePPND studies for biologics have replaced the former separate embryo-fetal and PPND study types. This work provides a retrospective analysis of pregnancy outcomes from 21 ePPND studies and group sizes of 14–24 animals per group. All studies reached the goal of at least six to eight infants on PND7, with overall losses ranging between 5 % and 45 %. Consistently, a group size of 14–24 maternal animals yielded more than six to eight infants on PND7. Therefore, it is suggested to reduce ePPND study group sizes from 20 to 14, yielding an animal number reduction of approx. 30 %.

## 1 Introduction

Selection of an appropriate group size for developmental toxicity studies has been challenging due to the spontaneous pregnancy losses (abortions and stillbirths) and infant losses in nonhuman primate (NHP) models, e.g. the long-tailed macaque (cynomolgus monkey). Typically, around 20 maternal animals per group have been used in pre- and postnatal development (PPND) and enhanced PPND (ePPND) studies. Pregnancy outcome in the cynomolgus monkey can be predicted statistically by using group-size-related normograms based upon variability of pregnancy success data experienced in over 60 studies in pregnant animals (Jarvis et al., 2010). Among these studies were mostly embryo-fetal development studies (Jarvis et al., 2010). Since ICH M3(R1) (2009) and ICH S6(R1) (2011), however, the predominant default Developmental And Reproductive Toxicology (DART) study type are ePPND studies. As per ICH S6(R1) (2011), it is recommended to start with group sizes of pregnant animals that yield six to eight live infants per study group on postnatal day 7 (PND7). For this work, pregnancy success and infant survival until PND7 were analyzed from control groups in 21 ePPND studies with respect to achieving the recommendations of ICH S6(R1) (2011).

**Figure 1:**
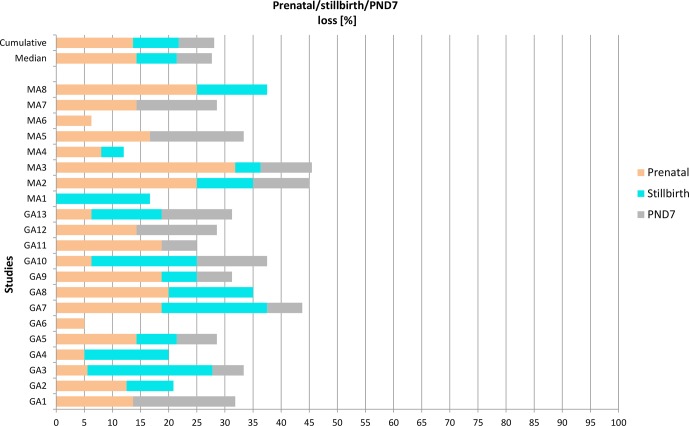
Prenatal loss, stillbirth, and postnatal loss until postnatal day 7 (PND7) in 21 ePPND Studies for Münster (GA1-13) and Madison (MA1-8). Group sizes at study start were 14–24 animals per group.

## 2 Material and methods

A total of 21 ePPND studies performed at Covance Münster or Madison were evaluated, comprising over 380 animals. All studies were approved by the animal welfare committee/Institutional Animal Care and Use Committee (IACUC), and husbandry was in accordance with EU guidelines (Council of Europe, 2007). The animals were housed in pairs or groups during the entire conduct of the ePPND studies, and were co-housed with their infants. Animals were fed certified lab diet for primates and a supplemental diet enriched with Vitamin D3, either once or twice daily; this was supplemented by fresh fruit and vegetables, and tap water was available ad libitum. A climate-controlled room with a minimum of eight air changes per hour had temperatures of 19 to 25 ${}^{\circ }$C in the Münster site and 20 to 26 ${}^{\circ }$C in the Madison site, and relative humidity was 30 %–70 % in the Madison site and 40 %–70 % in the Münster site. The light–dark schedule was 12:12, and additional UV light was provided following the first delivery. Pregnancy and infant data comprise the study time frame between gestation day 20 until PND7 from control/vehicle-treated animals. Group sizes ranged from 14–24 animals.

**Figure 2:**
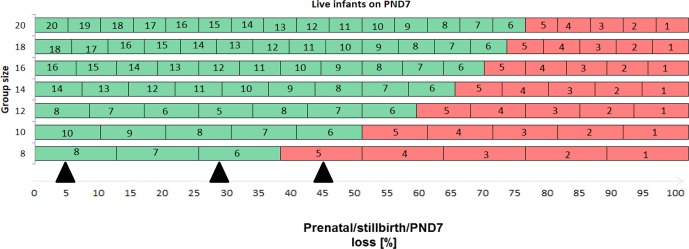
Relationship between group sizes within/above (green) or below (red) the recommended range of live infants on PND 7, pregnancy losses, and initial group size in ePPND studies. Triangles denote the observed minimal loss (5 %), maximal loss (45 %), and average/median loss (approx. 28 %) as described in text and depicted in Fig. 1. As per ICH S6(R1) (2011), it is recommended to start with group sizes of pregnant animals that yield six to eight live infants per study group on PND7. Group sizes of 12–14 animals will achieve that recommendation.

## 3 Results and discussion

ICH S6(R1) (2011) provides many study design recommendations resulting in considerably more standardized ePPND study layouts (Weinbauer et al., 2011, 2013) including the recommendation to plan studies such that six to eight live infants are available on PND7. Figure 1 shows the percentage of pre- and postnatal losses. The cumulative overall pregnancy loss rate was 28.1 %; the median overall pregnancy loss rate per study was 27.7 %. The individual data show a range of overall pregnancy loss rate per study of 5 % to 45 %. A group size of 14–24 yielded on average 13 live infants on PND7, with a range of 9–22 infants. Hence, group sizes of 14–24 maternal animals yielded always more than 6–8 infants on PND7. In Fig. 2, the loss rates are put in relation to achieving six to eight live infants on PND7. Even with the highest observed loss of 45 %, a group size of 14 pregnant animals would yield at least seven infants on PND7.

Jarvis et al. (2010) reported on 14 PPND studies, with six studies having group sizes of 14–24, five studies having group size of 12, and the remaining three studies had lower group sizes. For the group sizes of 14–24, numbers of 7–13 live infants were achieved by PND7, among which one study had 50 % (12 live infants at group size 24) overall loss, and one study had 56 % overall loss (7 live infants at group size of 16). Similar to the current dataset, a group size of 14 to 24 maternal animals consistently yielded at least six to eight infants on PND7. For the five PPND studies with a group size of 12 pregnant animals (Jarvis et al., 2010), 6, 8, 8, 8, and 10 live infants were available on PND7. Based on these empirical data, a group size of 12 would also be sufficient, but at maximal loss around 50 % could render a minimum infant numbers without a “comfort margin”.

**Figure 3:**
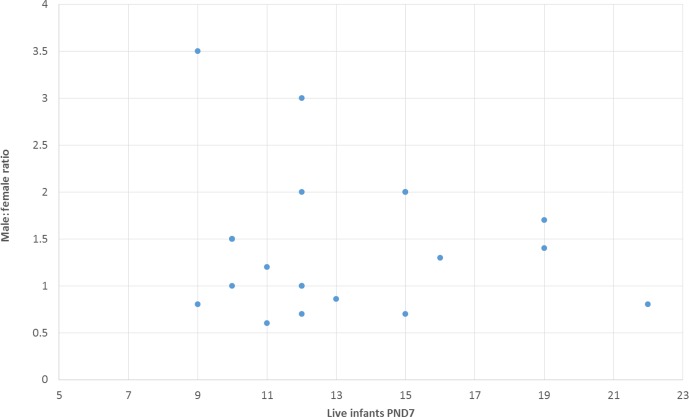
Comparison of male : female ratio versus number of live infants on PND7. It appears that the gender ratio is unrelated to the number of live infants (Pearson correlation coefficient: -0.14).

The male : female infant ratio on PND7 was on average 1.24 (median: 1.20) and ranged from 0.6 to 3.5. These ratios do not correlate to the number of infants on PND7 (Fig. 3). For example, gender ratio was either 0.8 or 3.5 in two studies with a group size of nine live infants. Importantly, albeit there is a wide scatter of male : female ratios across studies, a lower number of live infants on PND7 does not bear the risk of an imbalanced gender distribution.

Having less than six to eight live infants on PND7 in a test-item-exposed group as such does not imply an adverse effect. Such an effect would also depend on the infant numbers in the control group and would be based on an inspection of related normograms (Jarvis et al., 2010). Conversely, if an ePPND study starts with larger group sizes, a reduced number of infants available after PND7 can in fact indicate an adverse outcome even if that infant number exceeds six to eight live infants per group. Furthermore, it has been the experience in this laboratory, that normogram-based monitoring of an ePPND study can be used to enroll more animals in case that test-item-related increased incidence of abortions have occurred, and a concern arises that six to eight live infants may not be achieved on PND7.

ICH S6(R1) (2011) recommends minimal postnatal periods of infant observation for evaluation of T-cell-dependent antibody response (TDAR) testing and for testing of learning and memory aspects. This recommendation is driven by having infants of either appropriate body size for blood volume collection or a maturation status that allows for learning and memory testing. With regard to animal number, there are reports that a group size of eight animals for TDAR (Lebrec et al., 2011) and a group size of six to eight animals for lymphocyte immunophenotyping (Krejsa et al., 2013) provide acceptable statistical power for detecting alterations. It has also been demonstrated for learning and memory testing in juvenile animals that a group size of eight animals yields sufficient statistical power (Rose et al., 2015). In the current dataset, less than nine live infants per group in an ePPND study have not been encountered since ICH S6(R1) (2011) is effective. However, six to eight animals is considered sufficient for general toxicity studies. Hence, an initial group size of 14 animals per group should generally be sufficient if above infant testing parameters are required.

In conclusion, a group size of 14 pregnant animals per group is considered sufficient to predictably achieve six to eight live infants per study group on PND7 as recommended by ICH S6(R1) (2011).

## Supplement

10.5194/pb-7-1-2020-supplement The supplement related to this article is available online at: https://doi.org/10.5194/pb-7-1-2020-supplement.

## Data Availability

Data are provided as supplement for Figs. 1 and 3, and are uploaded. Figure 2 is not based on calculations.
