# Supplementary material for: Group size experiences with enhanced pre- and postnatal development studies in the long-tailed macaque (Macaca fascicularis)
Source: Primate Biol. 2020 Mar 11;7(1):1–4. doi: 10.5194/pb-7-1-2020 (PMC7096737; doi:10.5194/pb-7-1-2020)
Supplement: The supplement related to this article is available online at: https://doi.org/10.5194/pb-7-1-2020-supplement. [file pb-7-1-supplement.zip › pb-7-1-2020-supplement-title-page.pdf]

Supplement of Primate Biol., 7, 1–4, 2020
https://doi.org/10.5194/pb-7-1-2020-supplement
© Author(s) 2020. This work is distributed under
the Creative Commons Attribution 4.0 License.

Primate Biology
Open Access

*Supplement of*

# Group size experiences with enhanced pre- and postnatal development studies in the long-tailed macaque (*Macaca fascicularis*)

**C. Marc Luetjens et al.**

*Correspondence to:* Gerhard Weinbauer (gerhard.weinbauer@covance.com)

- pb-7-1-2020-supplement-title-page.pdf
- supplement data for Figure 1.xlsx
- supplement data for Figure 3.xlsx

The copyright of individual parts of the supplement might differ from the CC BY 4.0 License.
